# Investigating the Prevalence of Fungi in Diabetic Ulcers: An Under‐Recognised Contributor to Polymicrobial Biofilms

**DOI:** 10.1111/apm.70025

**Published:** 2025-04-22

**Authors:** Jontana Allkja, Ahmed Bakri, Bryn Short, Andrew Gilmour, Jason L. Brown, Abhijit M. Bal, Kelly J. M. Newby, Toby Jenkins, Rob D. Short, Craig Williams, Gordon Ramage

**Affiliations:** ^1^ Safeguarding Health Through Infection Prevention (SHIP) Research Group, Research Centre for Health, School of Health and Life Sciences Glasgow Caledonian University Glasgow UK; ^2^ School of Medicine, Dentistry and Nursing, College of Medical, Veterinary and Life Sciences University of Glasgow Glasgow UK; ^3^ Microbiology Department Glasgow Royal Infirmary Glasgow UK; ^4^ Pharmacy Department University Hospitals of Morecambe Bay NHS Foundation Trust UK; ^5^ Department of Chemistry The University of Sheffield Sheffield UK; ^6^ Department of Chemistry University of Bath Bath UK; ^7^ Microbiology Department, Lancaster Royal Infirmary University of Lancaster Lancaster UK

**Keywords:** chronic wound, diabetic foot ulcer, fungi

## Abstract

Diabetic foot ulcers (DFUs) are common complications for diabetic patients, often exacerbated by complex polymicrobial biofilm infections. While the majority of DFU studies are bacterial focused, fungi have also been identified. This study aims to investigate the prevalence of fungi in DFUs, as well as their potential role and influence on persistence and wound healing. Consecutive DFU swabs were collected from 128 patients (*n* = 349). Fungal positivity was assessed using enhanced culture and real‐time qPCR. Routine microbiology cultures were carried out as part of standard care in the clinics, and their results were then compared to our laboratory investigation. Routine and enhanced culture resulted in similar rates of fungal detection (~9%), whereas qPCR resulted in a higher rate of detection (31%). Notably, the predominant yeast *Candida parapsilosis* was present in ischaemic and penetrating bone wounds. These findings support existing evidence of fungal presence in DFUs. We demonstrated that routine diagnostic methods are sufficient for fungal detection, but enhanced culture methods allow for more precise fungal identification. Finally, while fungal presence does not appear to impact patient outcomes in our study, their role within these infections remains poorly understood, and further studies are needed to fully understand their relationship to the microbiome.

## 1 Introduction

Diabetic foot ulcers (DFU) represent a major challenge in the care of diabetic patients, severely impacting their quality of life. Biofilm‐associated infections play a key role in delayed healing, leading to severe outcomes like amputations. With an estimated cost of £962 million per annum, the financial burden of managing DFUs in the United Kingdom is vast [1].

Skin microbiota comprises bacteria and fungi in both diabetic and nondiabetic individuals [2]. However, while bacteria are recognised as the main pathogens responsible for DFU infections, fungi remain under‐investigated and under‐reported [3]. *Staphylococcus aureus* is one of the most frequently isolated species from wound infections and is considered a primary pathogen, with its ability to form biofilms being an important virulence factor [4, 5]. Another important bacterial pathogen often associated with wound infections is *Pseudomonas aeruginosa* [6] The majority of in vitro wound biofilm studies focus on these paradigm pathogens [7, 8]. However, a meta‐analysis of the literature by Malone et al. showed that chronic wounds are often characterised by polymicrobial interkingdom biofilms with a high tolerance to traditional antimicrobial treatments [9].

The Kalan group has shown the importance of the mycobiome in wound healing [10], though without careful mycological culture, it is difficult to demonstrate causality. Hence, modelling fungi within biofilms may provide a better understanding of the complexity of these infections and how to effectively manage them [11, 12, 13]. Studies of multispecies biofilms containing fungi have revealed an increased tolerance to treatment, reinforcing the argument for fungal consideration when developing chronic wound models and clinical intervention [14, 15].

In this study, we sought to investigate the prevalence of fungi within DFUs using culture‐based methods and targeted PCR and to assess their impact on clinical outcomes. The results of this study can help improve our understanding of fungal presence and impact in DFUs, as well as guide future research in biofilm wound model development and DFU management.

## 2 Materials and Methods

### 2.1 Collection of Clinical Samples

**FIGURE 1:**
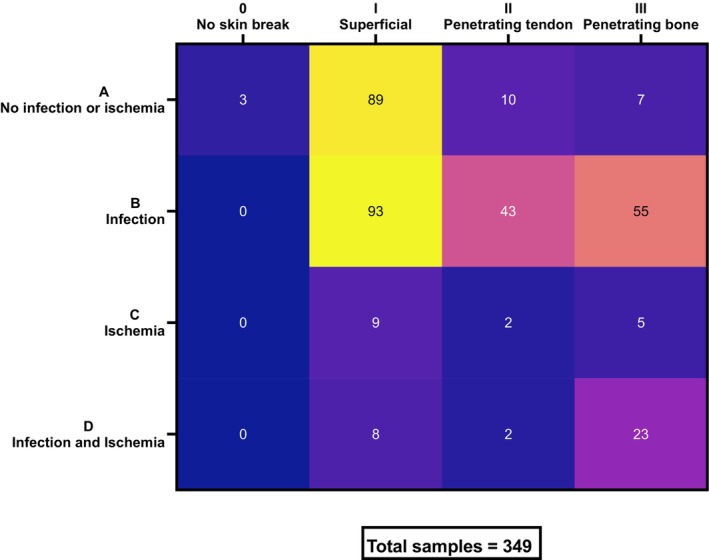
Wound swab classification according to the University of Texas classification system. Wounds are graded by depth: Grade 0 indicates pre or post ulcerative site, Grade 1 represents superficial wounds through the epidermis, Grade 2 wounds that penetrate to tendon or capsule and Stage 3 where deep ulceration penetrates to bone or joint [16] Wound stages are split into four: Nonischaemic clean wounds (A), nonischaemic infected wounds (B), ischaemic wounds (C) and infected ischaemic wounds (D) [16].

A total of 128 diabetic patients were recruited in this study from the Royal Lancaster Infirmary (RLI) and small community clinics in the Lancaster area who were attending for clinical assessment and required a diagnostic swab as part of their routine clinical care. Informed consent was obtained and swabs were obtained from the wound for routine culture and secondary processing by the academic team. Swabs of the entire region of the wound were taken according to the Royal Marsden Manual of Clinical and Cancer Nursing Procedures (https://www.rmmonline.co.uk/contents/procedures). Assessment of the location and the condition of the wound was recorded based on the University of Texas wound classification system [16]. Briefly, wounds were categorised according to Grades 0, I, II and III, which correspond to the wound depth, and also Stages A, B, C and D, which indicate the presence of infection or ischaemia (Figure 1). Swabs were collected from repeat visits where indicated, resulting in a total sample number of 349 wound swabs. These were classified according to their different grades and stages. Wound swabs were collected and kept in dry and glycerol vials to be used for molecular and culture work respectively. Control swabs were collected from air samples in the treatment room to account for environmental contaminants. Ethical approval was granted from HRA and Health and Care Research Wales (HCRW) (IRAS 293291).

### 2.2 Routine Microbiology Culture

As part of standard care practice, routine diagnostic microbiology culture was carried out at the Microbiology Department of Royal Lancaster Infirmary (University Hospitals of Morecambe Bay NHS Foundation Trust), using the UK Standards for Microbiology Investigations (SMIs) B 11 Investigation of swabs from skin and superficial soft tissue infections (https://www.rcpath.org/profession/publications/standards‐for‐microbiology‐investigations/bacteriology.html). For fungi, swabs are plated onto Sabouraud agar at 28°C–30°C for 7 days and observed daily.

### 2.3 Enhanced Fungal Culture

Enhanced culture was performed from 339 glycerol swabs. Ten swabs were not available for culture. The wound swabs were maintained at −80°C, thawed and vortex mixed. Samples were inoculated and spread across Sabouraud dextrose agar (SAB [ThermoFisher Scientific, UK]) supplemented with chloramphenicol (ThermoFisher Scientific, UK) and CHROMagar *Malassezia* (CHROMagar, France). All plates were incubated at 30°C and observed every alternate day for evidence of fungal growth until day 14. Subsequently, fungal isolates were purified on SAB for species identification using MALDI‐TOF at the Microbiology Department (Glasgow Royal Infirmary, UK).

### 2.4 ITS Real‐Time qPCR Screening

Fungal DNA was obtained from dry wound swabs using the MasterPure Yeast DNA extraction kit (LGC Biosearch Technologies, UK) according to the manufacturer's protocol with some modifications and additional steps. Prior to the heating step at 65°C for 15 min, samples were sonicated in 500 μL of lysis buffer provided in the kit. Once completed, the lysed cells were drawn out from the cotton swabs before continuing the steps as per manufacturer recommendations. Real‐time qPCR was performed using internal transcribed spacer (ITS) primers with the following sequences: ITS3 Uni F (5′‐TCGCATCGATGAAGAACGCAGC—3′) and ITS4 Uni R (5′—TCTTTTCCTCCGCTTATTGATATGC—3′) as previously described [13]. For qPCR, a mastermix containing Fast SYBR GreenER (Thermo Fisher Scientific, Paisley, UK), forward/reverse primers and UV‐treated RNase‐free water was prepared, to which extracted DNA was added. Standard curves for each strain were also included. The used thermal cycles were 50°C for 2 min, 95°C for 2 min, 40 cycles of 95°C for 3 s and 60°C for 30 s using Step‐One plus real‐time PCR machine and StepOne software V2.3 (Life Technologies, Paisley, UK). The cycle threshold value (Ct) was used to determine the colony forming equivalence (CFE) per millilitre by comparing it to the standard curve generated from *C. albicans* SC5314 with an efficiency of > 90%.

### 2.5 Statistical Analysis

All statistical analysis and graph production were performed using GraphPad Prism (Version 8.4.3; GraphPad Software Inc., La Jolla, CA).

## 3 Results

### 3.1 Fungi Are Prevalent in DFUs

#### 3.1.1 Classification of Samples

The 349 DFU swabs collected were classified according to the University of Texas wound classification system. As shown in Figure 1, the majority of the samples collected fell into the categories of superficial (Grade I) and either noninfected (Stage A) or infected (Stage B). A limited number of collected samples were from ischaemic (Stage C or D) and deeper wounds (Grade II or III). Moreover, Figure S1 in the supplementary file shows the distribution of wound swabs collected, separated into RLI samples (A) and community samples (B). Most of the samples were collected from RLI, and from the samples collected from community clinics, the overwhelming majority fall into the superficial (Grade I) and noninfected (Stage A) categories.

#### 3.1.2 Assessment of Fungal Prevalence in DFU Samples

**FIGURE 2:**
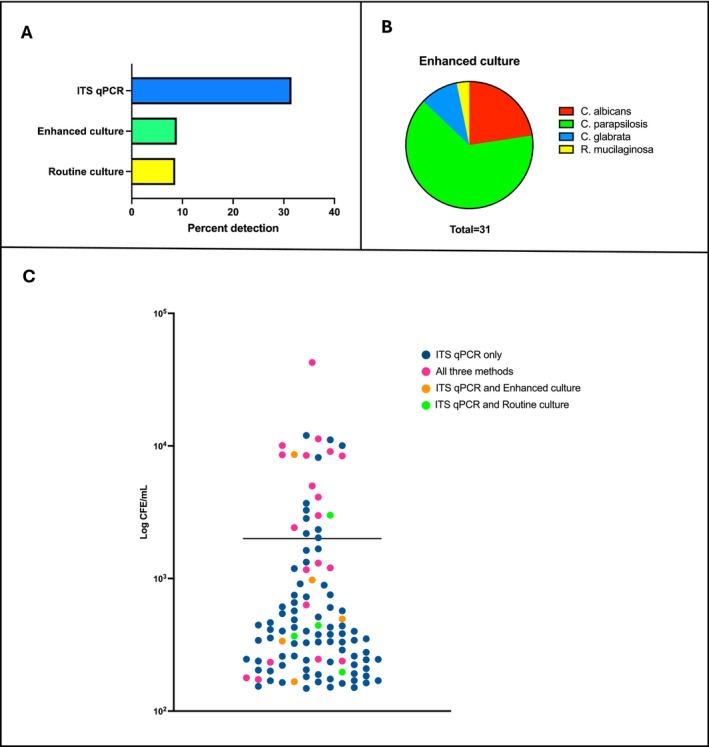
Fungal prevalence in DFU swabs. A. Percent detection of fungal presence separated by method. B. Identification of fungal species isolated through the enhanced culture method. **C**. Quantification of fungal load by ITS qPCR presented as CFE/mL. Individual value plot points were colour coded according to detection by different methods: Blue—detected by ITS qPCR alone, pink—detected by all three methods, orange—detected by ITS qPCR and enhanced culture, green—detected by ITS qPCR and routine culture. Ten samples were excluded from the graph as fungal presence was detected by either one or both culture methods, but not ITS qPCR. Kruskal–Wallis test was performed for multiple comparisons of detection rates. Significant difference at *p* < 0.05.

Table 1 and Figure 2 summarise data for samples where fungi were detected by each of the described methods. An extended version of this table includes data for all 349 samples, as well as information on wound classification, outcome at 3 months and the anatomical location where it was collected (Table S1). Figure 2A shows the percent detection of fungal presence according to each method. Quantitatively, no significant differences (*p* > 0.05) were observed between the two culture methods, as 30 swabs (8.6%) were positive for fungal growth in the routine culture reports and 31 swabs (8.9%) were positive in the enhanced culture method. Ten samples were positive for fungal growth with the enhanced culture method but not with the routine culture method. Nine samples were positive for fungal growth in the routine culture reports but not with the enhanced culture method. Using the more sensitive ITS qPCR method, fungal DNA was detected in 110 of the DFU swabs (31.5%). Ten samples were positive by either routine or enhanced culture, or both, but negative for ITS qPCR.

**TABLE 1:** **Fungal positive (FP) samples for routine culture, enhanced culture and ITS qPCR**. Fungal negative (FN) samples for all three methods are not included in the table. Routine culture results provided by the diagnostic laboratories report for bacterial species have been categorised as Gram‐positive, Gram‐negative or Others. N/A—not available. NG‐ No colony growth in culture.

Sample ID	Routine culture				Enhanced culture		
	Fungi	Gram positive	Gram negative	Others	Colony isolation	MALDI‐TOF	ITS qPCR
2_2	*C. albicans*		*Enterics*	Mixed skin	FP	*C. albicans*	FP
2_3	*C. albicans*	*S. aureus*	*Coliforms*		FP	*C. albicans*	FP
2_4	*C. albicans*	*S. aureus*	*Enterics*	Anaerobes	FP	*C. albicans*	FP
2_5	*C. albicans*	*S. aureus*	*Coliforms*		FP	*C. albicans*	FP
2_6	*C. albicans*	*S. aureus*		Anaerobes	FN		FN
2_7	FN	*S. aureus*	*Enterics*		FP	*C. albicans*	FP
2_8	FN	*S. aureus*	*Coliforms*	Anaerobes	FN		FP
2_9	FN	*S. aureus*	*Enterics*	Anaerobes, mixed skin	FN		FP
5_5	FN			Mixed skin	FN		FP
5_6	FN			Mixed skin	FN		FP
7_1	FN	*MRSA*			FN		FP
10_1	*Candida sp*	*S. aureus*			FP	*C. parapsilosis*	FP
10_2	*Candida sp*	*S. aureus*			FP	*C. parapsilosis*	FN
10_6	FN		*Coliforms*		FN		FP
10_7_b	FN		*Enterics*		FN		FP
10_8_a	FN		*Enterics*		FN		FP
10_11_a	FN		*Enterics*		FP	*C. parapsilosis*	FP
10_11_b	*Candida sp*		*Coliforms*	Mixed skin	FN		FP
11_4	FN			Mixed skin	FN		FP
12_1	FN	*S. aureus*			FN		FP
15_1	*C. albicans*		*Enterics, Pseudomonas species*		FN		FP
16_1	FN		*P. aeruginosa*	Anaerobes	FN		FP
17_1	FN		*Enterics, Pseudomonas species*		FN		FP
17_2	*Candida sp*	*Enterococcus*	*Pseudomonas species*		FP	*C. glabrata*	FP
17_3	*Candida sp*		*Pseudomonas species*	Mixed skin	FN		FN
18_1	*C. albicans*	*Enterococcus*		Mixed skin	FN		FP
18_2	*Candida sp*		*Coliforms*	Mixed skin	FP	*C. glabrata*	FP
18_4_b	*Candida sp*		*Enterics*		FP	*C. glabrata*	FP
19_2	FN			Mixed skin	FN		FP
22_3	FN			Mixed skin	FP	*R. mucilaginosa*	FP
23_3	*Candida sp*		*Enterics*		FN		FN
24_2	*Candida sp*			Mixed skin	FP	*C. parapsilosis*	FP
24_4	*Candida sp*	*S. aureus*	*Coliforms*		FP	*C. parapsilosis*	FP
27_2	FN			Mixed skin	FN		FP
27_3	FN	*S. aureus*	*Enterics*		FN		FP
27_4	FN	*S. aureus*	*Enterics*		FN		FP
27_5	FN		*Enterics, Pseudomonas species*		FN		FP
27_6	FN		*Enterics*		FN		FP
28_4	FN	*MRSA*	*Enterics*		FP	*C. parapsilosis*	FN
34_1	FN	*S. aureus*	*Coliforms*	Anaerobes	FN		FP
38_6	FN			Mixed skin	FN		FP
39_1	FN	*S. aureus*	*Coliforms*		FN		FP
45_1	FN		*Coliforms*		FN		FP
46_2	FN		*Coliforms, Pseudomonas species*		FN		FP
47_1	FN			Mixed skin	FN		FP
54_1	FN			NG	FN		FP
56_2	FN	*S. aureus*			FN		FP
57_1	FN		*Enterics*		FN		FP
57_2	FN	*Group C Strep*	*Enterics*		FN		FP
57_3	FN		*Enterics*	Mixed skin	FN		FP
57_5	FN	*Group G Strep*		Mixed skin	FN		FP
57_7_a	*C. albicans*	*MRSA*	*Enterics*		FN		FP
57_7_b	FN	*MRSA*			FP	*C. albicans*	FP
57_8	FN	*MRSA*			FN		FP
57_11	FN		*Enterics*		FN		FP
60_2	FN		*Coliforms*		FN		FP
64_2	N/A	N/A	N/A	N/A	FN		FP
65_1	FN	*S. aureus*	*Coliforms*		FN		FP
65_2	FN	*S. aureus*			FN		FP
65_7	FN			Mixed skin	FN		FP
65_8	FN	*S. aureus*			FN		FP
67_1	FN	*S. aureus*			FN		FP
68_2	FN	*S. aureus*			FN		FP
70_1	FN	*S. aureus*			FN		FP
71_1	FN			NG	FP	*C. parapsilosis*	FP
73_1	FN	*S. lugdunensis*			FN		FP
74_1	FN		*Coliforms bacilli*		FN		FP
75_1	FN	*S. aureus*			FN		FP
76_1	FN		*Enterics*		FN		FP
76_2	FN			Mixed skin	FN		FP
77_1	FN			NG	FN		FP
79_1	FN	*S. aureus*			FN		FP
80_3	FN		*Coliforms*	Mixed skin	FN		FP
84_1	FN		*Coliforms*		FN		FP
85_3	FN		*Enterics, Pseudomonas species*		FP	*C. parapsilosis*	FN
85_5	FN			Mixed skin	FN		FP
86_1	FN	*S. aureus*			FN		FP
88_1	FN	*S. aureus*			FN		FP
89_1	FN		*Coliforms*	Mixed skin, Anaerobes	FN		FP
90_1	FN	*S. aureus*			FN		FP
91_2	FN			No signifcant growth	FN		FP
92_2	FN		*Enterics*		FN		FP
93_1	FN	*S. aureus, Group B Strep*			FN		FP
93_3	FN	*S. aureus, Group B Strep*			FN		FP
93_4	FN	*Group B Strep*		Mixed skin	FN		FP
93_6	FN	*S. aureus, Group B Strep*			FN		FP
93_7	FN	*S. aureus*	*Enterics*		FN		FP
93_8	FN		*Coliforms bacilli*	Mixed skin	FN		FP
93_12	FN			Mixed skin	FN		FP
93_13	FN			Mixed skin	FN		FP
95_2	FN			Mixed skin, Anaerobes	FN		FP
97_2	FN			Mixed skin, Anaerobes	FN		FP
97_3	FN			Mixed skin, Anaerobes	FN		FP
97_4	FN		*Coliforms bacilli*	mixed skin	FN		FP
101_1	*Candida sp*		*Enterics*	Mixed skin	FP	*C. parapsilosis*	FP
102_1	FN			Mixed skin	FP	*C. parapsilosis*	FP
102_3	*Candida sp*		*Enterics*		FP	*C. parapsilosis*	FN
102_4	*Candida sp*		*Enterics*		FP	*C. parapsilosis*	FP
103_1	FN	*S. aureus*			FP	*C. parapsilosis*	FN
105_1	*C. albicans Candida sp*		*Enterics*		FN		FN
107_1	FN			NG	FN		FP
109_1	FN			*Mixed skin*	FN		FP
111_3	*Candida sp*			Anaerobes	FP	*C. parapsilosis*	FP
111_4	*Candida sp*			Anaerobes, mixed skin	FP	*C. parapsilosis*	FP
111_6	*Candida sp*	*S. aureus*		Anaerobes	FP	*C. parapsilosis* ^*#*^	FP
112_2_a	*Candida sp*		*Pseudomonas species*		FP	*C. parapsilosis*	FP
112_2_b	FN		*Pseudomonas species*	Anaerobes	FN		FP
112_3	FN		*Coliforms, Pseudomonas species*	Anaerobes	FP	*C. parapsilosis*	FP
116_4	FN		*Enterics, Pseudomonas species*		FN		FP
117_5_a	FN		*Enterics*		FN		FP
117_6	FN		*Enterics, Pseudomonas species*		FN		FP
120_1	*C. albicans*		*Enterics*		FP	*C. albicans*	FN
122_1	FN			Anaerobes, mixed skin	FN		FP
122_3_a	FN			Mixed skin	FN		FP
122_3_b	FN	*S. aureus*			FN		FP
123_1	FN			Mixed skin	FN		FP
123_4	*Candida sp*		*Enterics*		FP	*C. parapsilosis*	FP
125_2	FN			Mixed skin	FP	*C. parapsilosis*	FN
126_1	FN			Mixed skin	FN		FP
128_1	*C. albicans*		*Enterics*	Mixed skin	FN		FN

Figure 2B shows the distribution of fungal species identified by enhanced culture using MALDI‐TOF. Qualitatively, *Candida parapsilosis* was the most prevalent (20 samples) followed by *C. albicans* (7 samples) and *C. glabrata* (3 samples). *Rhodotorula mucilaginosa* was the only non‐*Candida* species and was isolated from a single sample. Interestingly, when looking at specific patients with repeat visits and repeat fungal presence in the wound, the same fungal species appears each time (Table 1). For example, *C. albicans* appears in all enhanced and routine culture results for patient 2. Whereas for patient 10, only *C. parapsilosis* is detected in multiple visits by enhanced culture. This could suggest that a particular fungal species preferentially colonises specific patients; however, given our limited enhanced culture results, further investigation would be required.

Routine culture discriminated the fungal growth into *C. albicans* or *Candida* species (Table 1). The routine culture reports for *C. albicans* agree with *C. albicans* identification through MALDI‐TOF. Conversely, samples identified as *C. parapsilosis* or *C. glabrata* through MALDI‐TOF were classified as *Candida* species, which shows a limited scope to identify non‐*albicans Candida* species through routine culture methods.

Fungal load values were also quantified through ITS qPCR methodologies. These are shown in the individual value plot in Figure 2C and are expressed as colony‐forming equivalents per millilitre, with a mean value of 2 × 10^3^ CFE/mL. Individual value plot points were color coded according to detection by different methods. Ten samples are not shown in the graph due to nondetectable fungal DNA by ITS qPCR. Based on these results, fungal detection by either culture method appears to be unrelated to fungal DNA concentrations. This suggests that while qPCR is a more sensitive method, culture methods are also able to detect fungal presence at low concentrations. A direct comparison of CFU/mL versus CFE/mL for fungal positive samples detected with both enhanced culture and ITS qPCR is shown in Figure S2. The figure shows that CFE/mL values are higher than CFU/mL (*p* = 0.0027). Interestingly, in the samples with the lowest CFE/mL values, the corresponding CFU/mL values are higher. However, it is important to keep in mind that the swabs used for fungal DNA extraction and the ones for culture were kept under different conditions, dry and glycerol collection tubes respectively. This could be affecting the rate of recovery for each method. When this is considered, the disparities in detection by either of the three methods for example, detection with culture methods but not qPCR, indicate that while more sensitive methods like ITS qPCR have a higher rate of detection overall, they are not infallible.

### 3.2 Impact of Fungal Presence on DFUs

#### 3.2.1 Characterisation of Interkingdom Relationships

**FIGURE 3:**
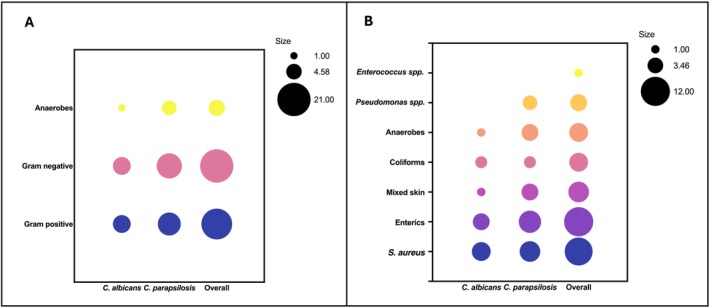
Interkingdom relationships between fungi detected in enhanced culture and bacterial species reported by routine culture. Data represented as bubble plots. Bubble size corresponds to the number of samples A. Bubble plot of co‐isolation of *C. albicans* , *C. parapsilosis* and overall fungi with bacteria grouped as Gram negative, Gram positive and Anaerobes. B. Bubble plot of co‐isolation of *C. albicans* , *C. parapsilosis* and overall fungi with bacteria grouped as reported in routine culture results shown in Table 1. Two‐way ANOVA with a post hoc Tukey multiple comparison test was performed. Significant difference at *p* < 0.05.

Fungi are part of polymicrobial interkingdom communities composed of a variety of bacterial species. We investigated the association between the different fungal species we isolated with enhanced culture and the bacterial species reported in routine culture. Figure 3 presents the rates of co‐isolation of different bacterial species/groups with fungi identified through MALDI‐TOF in enhanced culture, based on the data available in Table 1. Rates have been calculated for different types of fungal species: *C. albicans, C. parapsilosis* and all fungi. Figure 3A groups bacteria as Gram negative (*Enterics, Pseudomonas* spp. *and Coliforms*), Gram positive (*S. aureus, Enterococcus* spp. and skin flora) and anaerobes. Figure 3B shows the rates for each of these individual groups as reported in routine culture (Table 1).

In Figure 3A, there is a greater association between fungi and Gram‐negative bacteria (21 out of 31) compared to Gram‐positive (18 out of 31), though not statistically significant (*p* > 0.05). When looking at the more specific groups in Figure 3B, these seem to be mostly enterics (12 samples) and *S. aureus* (11 samples). Similar ratios could be observed when looking at *C. albicans* and *C. parapsilosis* specifically. However, one interesting result is the overwhelming association between anaerobes and *C. parapsilosis* compared to *C. albicans* . Out of six samples containing anaerobes, five of them were co‐isolated with *C. parapsilosis* .

#### 3.2.2 Association Between Fungal Prevalence and Wound Classification

**FIGURE 4:**
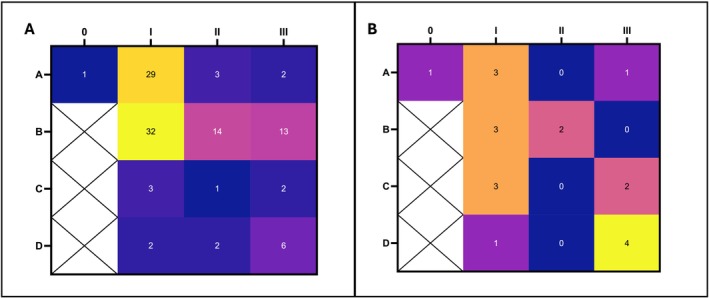
Distribution of fungal‐positive samples according to wound classification. Data represented a 4 × 4 matrix, y‐axis represented wound stage, x‐axis represented wound grade. **A**. Distribution of ITS qPCR fungi positive samples according to wound classification. B. Distribution of *C. parapasilosis* samples isolated by enhanced culture according to wound classification.

To further understand the potential impact of fungal presence, we decided to evaluate the distribution of ITS qPCR fungal‐positive samples according to the wound type, as summarised in Table S1. These data were summarised and compiled into a 4 × 4 matrix, where wound stage is plotted on the y‐axis and wound grade on the x‐axis (Figure 4A). The majority of samples fall into Stage B (infected) and Grade I (superficial). Additionally, considering the low number of available samples in ischaemic (Stages C and D) and deep penetrating (Grades II and III) (Figure 1), a large proportion of these were scored as positive for fungal presence. Considering the frequency of *C. parapsilosis* in our enhanced culture results, we reviewed the distribution of these samples according to wound classification (Figure 4B). A larger proportion of these samples fall into the Stage D, Grade III group. In fact, the majority of fungal‐positive samples at Stages C and D and Grades II and III appear to be related to the presence of *C. parapsilosis* , albeit with low overall numbers.

#### 3.2.3 Impact of Fungal Presence on Healing Outcomes

**FIGURE 5:**
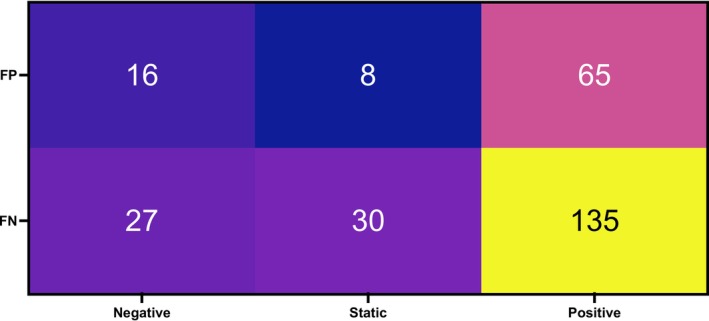
Impact of fungal presence detected by ITS qPCR on wound healing outcomes. Wound healing outcomes in Table S1 are grouped as positive (healed, still present and improved), negative (amputated, still present but worsened) and static (still present and static). Data arranged according to fungal negative (FN) and fungal positive (FP) result of ITS qPCR assessment. Samples with missing outcomes (64) and deceased patients (4) were removed from assessment. Two‐way ANOVA with a post hoc Tukey multiple comparison test was performed. Significant difference at *p* < 0.05.

Healing outcomes are another important factor to consider when assessing the impact of fungal presence on DFUs. Table S1 lists the outcomes at 3 months as reported by the clinical team. For our assessment, we grouped them as positive outcomes (‘healed’ and ‘still present, but improved’), negative outcomes (‘amputated’ and ‘still present, but worsened’) and static (‘still present, but static’). Samples reporting ‘patient deceased’ (4 samples) and ‘missing’ (64 samples) were excluded from the analysis. The total sample number assessed was 281. Impact on outcome was assessed according to ITS qPCR results as grouped as fungal positive (FP) and fungal negative (FN). Results are summarised in the heatmap in Figure 5, where samples categorised according to fungal presence (y‐axis) and outcome (x‐axis). No significant difference (*p* > 0.05) in outcome can be seen depending on fungal presence. For positive outcomes, 33% of samples are fungal positive, compared to 37% of samples for negative outcomes.

However, it is important to remember that these results only reflect the impact based on fungal ITS detection. The effect of fungi detected by culture‐based methods could not be assessed due to missing information on patient outcomes, particularly for *C. parapsilosis* samples.

## 4 Discussion

DFUs are at high risk of developing infection due to exposure to a plethora of microbes either from the skin or the environment, combined with a lowered immune response due to the patient's diabetic status [17]. Unlike bacteria, the clinical importance of fungi is often dismissed or neglected. However, various studies have identified many different species of fungi from DFU swabs, such as *Candida* species and filamentous moulds, which include dermatophytes and other ubiquitous moulds like *Aspergillus* and *Fusarium* species [18, 19, 20].

We performed an observational study of fungal prevalence in DFU swabs collected from 128 patients over several visits to the RLI and community‐based clinics in the Lancaster area. Routine microbiology culture reports performed as part of patient standard care detected fungal presence in 8.6% of samples. We compared this to the enhanced culture method, which reported similar rates of 8.9%, and ITS qPCR, which reported the presence of fungal DNA in 31.5% of samples. Encouragingly, other studies have shown similar results when using molecular techniques. For example, in the study by Kalan et al., they found that about 70% of their DFU samples were positive for fungal DNA when sequenced using the ITS1 primers [19]. In a separate study performed in a much larger cohort of wound specimens from various wound types, including DFUs, the incidence was reported to be at 23%, comparable to our own [21].

Several samples showed disparities in detection between the methods, and while more often samples were detected with qPCR and not either culture method, occasionally the opposite scenario occurred (Table 1). Moreover, when looking at fungal load based on ITS qPCR (Figure 2C), detection with multiple methods appears unrelated to CFE/mL values and is scattered from the lower to the higher concentrations. A possible explanation for this could be due to the fact that a single fungal cell may contain multiple copies of rDNA and these numbers can also vary greatly between species [22]. For example, an *in silico* study showed that there can be a range of 14–1442 copies of rDNA across 91 fungal taxa [23]. Hence, molecular data need to be interpreted with caution, as this variability in rDNA could be affecting the ITS load recovered. This could mean that while for certain samples fungal load for DNA may appear low, the number of culturable cells is high enough for culture methods to detect them and vice versa, while fungal load may appear high the number of culturable cells may be too low for detection with culture methods. Hence, as our data suggest for the purposes of the traditional culture method, it remains a reliable gold standard.

When compared with the more specific enhanced culture method, the rate of detection by routine culture was not significantly different. However, the main difference was in the added benefit of identifying fungal species. While *C. albicans* was identified correctly with both methods, in the case of *non‐albicans Candida* species, the routine culture method was designed to report them as *Candida* species (Table 1). Our findings reported an overwhelming prevalence of *C. parapsilosis* in the wounds compared to the other *Candida* species (Figure 2B). This is important considering the recent addition of *C. parapsilosis* as a high‐priority fungal pathogen by WHO in 2022 [24]. It is commonly found on the skin and in the hospital environment, and in a recent report, *C. parapsilosis* resistant to fluconazole was identified as an emerging pathogen [25]. Moreover, when we performed some additional experiments on our fungal isolates (detailed in Supporting Informations), the *C. parapsilosis* isolates were consistently more tolerant to traditional antifungals (fluconazole, caspofungin and amphotericin B) in both planktonic and biofilm states compared to the *C. albicans* isolates (Table S2).

In the study by Kalan et al. mentioned above, no consistent patterns of fungal species across the subjects were found; nevertheless, *Candida* species were the most common, with *C. albicans* being the most frequent isolate [19]. Dowd and colleagues showed that DFUs were predominately colonised by *C. parapsilosis*, in comparison to venous leg ulcers and unhealing surgical wounds, which were dominated by *C. albicans* [26]. None of our wound samples grew filamentous moulds, as reported in other studies [7, 22, 27], despite prolonged incubation to facilitate the detection of moulds such as *Aspergillus*, *Fusarium* and *Cladosporium*, and slower‐growing dermatophytes. The only non‐*Candida* species recovered was the yeast *R. mucilaginosa* . It is important to note that the culture plate could be contaminated by fungi at any point during the incubation process, especially if the plates were incubated for a prolonged period, and careful consideration should be made on whether growth represents true presence or contamination.

It is important to remember that fungi do not exist in isolation; they are part of a larger polymicrobial community which includes a variety of bacterial species. In our study, we looked at the co‐isolation of fungi detected by enhanced culture with the bacteria reported by routine culture in the clinics. Similar rates of associations were observed between Gram‐negative and Gram‐positive bacteria. When looking at the more specific species/groups, Enterics and *S. aureus* were the most prevalent. The relationship of *Candida* species with enteric‐related bacteria has been similarly reported in another study [27]. This may not be surprising as their co‐existence has also been described in the gastrointestinal tract [28]. Moreover, the synergistic relationship between *C. albicans* and *S. aureus* has been extensively documented in the literature. Studies have shown that *C. albicans* plays a role in the growth of *S. aureus* biofilms by way of providing surface area for the bacteria to attach, allowing for further dissemination into the host [29, 30]. An interesting observation was the relationship between *C. parapsilosis* and anaerobes (Figure 3). While few studies are available on either of these organisms, literature suggests that *C. parapsilosis* has similar behavioural traits to *C. albicans* under hypoxic conditions [31]. Studies performed primarily on oral polymicrobial biofilms have shown that *C. albicans* can support the growth of obligate anaerobes under nonanaerobic conditions [32]. This suggests a similar role for *C. parapsilosis* in facilitating anaerobic species growth within DFUs.

The spread of fungal‐positive samples according to their wound type, as per the University of Texas Classification Scheme [16], which allows the clinician to document the depth of the ulcer and determine the presence of infection and/or ischaemia was analysed. NICE guidelines [33] indicate the use of the University of Texas or SINBAD to classify diabetic foot ulcers. These systems are an important tool to aid clinicians in the management and guidance of appropriate treatments for optimal patient outcomes. The majority of samples fell in the infected (Stage B) and superficial (Grade I) categories. A large number of samples were also in the noninfected (Stage A) category. This could potentially suggest that the presence of fungi does not play a role in determining wound severity. This is further supported when looking at the impact of fungal presence on outcomes; we did not find any significant differences between positive and negative outcomes. However, as we have speculated previously, fungi being present in an interkingdom environment may support bacterial growth and inadvertently synergise negative clinical outcomes [34]. Interestingly, considering the small number of samples available for ischaemic and deep penetrative wounds, a majority of them appear to have fungal presence. A large proportion of this appears to be attributable to *C. parapsilosis* , which could also be associated with its relationship with anaerobes. Similarly, other studies have identified *C. parapsilosis* as the most prevalent species isolated from deep tissue of diabetic lower limb wounds [18].

However, it is important to remember that these results should be interpreted with caution. While crucial to wound care, the University of Texas Classification has its limitations. It can be subjective and often undermines the severity of the wound. For instance, different clinical professions may classify the same wound differently due to experience, lack of experience or being unfamiliar with the system [35]. Another drawback of this classification system is its limited scope, as it does not consider the site, size and neuropathy of diabetic foot ulcer. This is important to know, as this can give a greater picture of the wound and the patient's diabetic‐related complications [35]. Hence, further studies employing a less subjective classification scheme and focusing on more severe, deeper wounds could provide a more in‐depth look into the role of fungi in DFU.

Overall, this study strengthens the evidence of fungi within DFUs and highlights *Candida* species as an important aetiology in the chronic wound biofilm. The interkingdom biofilm environment has implications for clinical management and poses the question as to whether consideration should be given to antifungals in the management of wounds. We propose that greater emphasis is placed on culture and identification of fungi within clinical medicine to develop a more structured epidemiological understanding of fungi in DFUs.

## Conflicts of Interest

The authors declare no conflicts of interest.

## Supporting information


**Figure S1.** Wound swab classification according to the University of Texas classification system. A. Wound samples collected from the Royal Lancaster Infirmary B. Wound samples collected from community clinics. Wounds are graded by depth: Grade 0 indicates pre or post ulcerative site, Grade 1 represents superficial wounds through the epidermis, Grade 2 wounds that penetrate to tendon or capsule and Stage 3 is where deep ulceration penetrates to bone or joint. Wound stages are split into four: nonischaemic clean wounds (A), nonischaemic infected wounds (B), ischaemic wounds (C) and infected ischaemic wounds (D).
**Figure S2.** Pairwise comparison of CFU/mL versus CFE/mL for fungal positive samples for enhanced culture and ITS qPCR. Mann–Whitney test was performed on log‐transformed values. Significant differences at *p* < 0.05.
**Table S1.** Summary of fungal prevalence investigation results and patient clinical information. The presence of fungi has been categorised as fungal negative (FN) and fungal positive (FP). Routine culture results provided by the diagnostic laboratories report for bacterial species have been categorised as Gram‐positive, Gram‐negative or Others. N/A—not available. NG—No colony growth in culture. RLI—Royal Lancaster Infirmary.
**Table S2.** Planktonic MIC (sMIC) and sessile MIC (sMIC) of fungal species isolated from wound swabs, against three conventional antifungals: Fluconazole (FZ), caspofungin (CAS) and amphotericin B (AMB). Values represent median from three replicates. Growth inhibition was assessed visually, except for fluconazole which was read by a spectrophotometer at 530 nm for 50% inhibition due to the trailing effect. Symbols^#^ and^†^ indicate smooth and wrinkled phenotype of *C. parapsilosis* complex isolates respectively.

## Data Availability

Primer sequences can be found here: https://www.glasgowbiofilms.co.uk/. Raw data is available upon request.
