# Supplementary material for: Investigating the Prevalence of Fungi in Diabetic Ulcers: An Under‐Recognised Contributor to Polymicrobial Biofilms
Source: APMIS. 2025 Apr 22;133(4):e70025. doi: 10.1111/apm.70025 (PMC12015384; doi:10.1111/apm.70025)
Supplement: Supplementary file 1 — Figure S1. Wound swab classification according to the University of Texas classification system. A. Wound samples collected from the Royal Lancaster Infirmary B. Wound samples collected from community clinics. Wounds are graded by depth: Grade 0 indicates pre or post ulcerative site, Grade 1 represents superficial wounds through the epidermis, Grade 2 wounds that penetrate to tendon or capsule and Stage 3 is where deep ulceration penetrates to bone or joint. Wound stages are split into four: nonischaemic clean wounds (A), nonischaemic infected wounds (B), ischaemic wounds (C) and infected ischaemic wounds (D). Figure S2. Pairwise comparison of CFU/mL versus CFE/mL for fungal positive samples for enhanced culture and ITS qPCR. Mann–Whitney test was performed on log‐transformed values. Significant differences at p < 0.05. Table S1. Summary of fungal prevalence investigation results and patient clinical information. The presence of fungi has been categorised as fungal negative (FN) and fungal positive (FP). Routine culture results provided by the diagnostic laboratories report for bacterial species have been categorised as Gram‐positive, Gram‐negative or Others. N/A—not available. NG—No colony growth in culture. RLI—Royal Lancaster Infirmary. Table S2. Planktonic MIC (sMIC) and sessile MIC (sMIC) of fungal species isolated from wound swabs, against three conventional antifungals: Fluconazole (FZ), caspofungin (CAS) and amphotericin B (AMB). Values represent median from three replicates. Growth inhibition was assessed visually, except for fluconazole which was read by a spectrophotometer at 530 nm for 50% inhibition due to the trailing effect. Symbols# and† indicate smooth and wrinkled phenotype of C. parapsilosis complex isolates respectively. [file APM-133-0-s001.docx]

**Supplementary material:**

**Investigating the prevalence of fungi in diabetic ulcers: an under-recognised contributor to polymicrobial biofilms**

**Supplementary methods**

**Planktonic and Sessile MIC**

Antifungal susceptibility profiles were tested for the fungal isolates in their planktonic and sessile (biofilm) state. Three conventional antifungals: Fluconazole (Sigma- Aldrich, Gillingham, UK), Caspofungin (Sigma- Aldrich, Gillingham, UK) and Amphotericin B (Sigma- Aldrich, Gillingham, UK) were prepared in water or DMSO to a stock concentration according to the manufacturer’s instruction. All three antifungals were serially diluted in a flat bottom 96 well plate, in RPMI-1640 medium to give a concentration range from 128 – 0.25 μg/mL.

For planktonic MIC testing, the fungal inoculum was standardised to 2 x 10^4^ cells/mL in RPMI-1640 and added to the prepared antifungal plate. The final concentrations of antifungals tested ranged from 64 – 0.0125 μg/mL. Following a 24-h incubation at 37℃, the MIC was visually examined as the lowest concentration with no visible growth. A trailing effect was observed for some isolates when tested with Fluconazole and these were further examined for 50 % inhibition using a spectrophotometer at 530 nm.

For sessile MIC testing, biofilms were grown as before, and the antifungal effect was measured as previously described (15). Serially diluted antifungals ranging from 128 – 0.25 μg/mL were added to the preformed biofilm. RPMI-1640 alone was used as a negative control. Plates were incubated for 24-h at 37℃, the antifungals were then removed and replaced with XTT (2,3‐bis(2‐Methoxy‐4‐Nitro‐5‐Sulfophenyl)‐2H‐Tetrazolium‐5‐Carboxanilide; ThermoFisher Scientific, UK) with 1 μM menadione for 2 hours, incubated at 37℃ in the dark. Once developed, 75 μL of the supernatant were transferred to a fresh 96-well microtiter plate and absorbance was read at a wavelength of 492 nm with a FLUOstar Omega Plate Reader (BMG Labtech, Aylesbury, UK) to determine 90 % biofilm inhibition.

**Supplementary figures**

**A**

**B**

**Figure S1. Wound swab classification according to the University of Texas classification system.** **A.** Wound samples collected from the Royal Lancaster Infirmary **B.** Wound samples collected community clinics. Wounds are graded by depth: grade 0 indicates pre or post ulcerative site, grade 1 represents superficial wounds through epidermis, grade 2 wounds that penetrate to tendon or capsule and stage 3 where deep ulceration penetrates to bone or joint. Wound stages are split into 4: non ischaemic clean wounds (A), non-ischaemic infected wounds (B), ischaemic wounds (C) and infected ischaemic wounds (D).


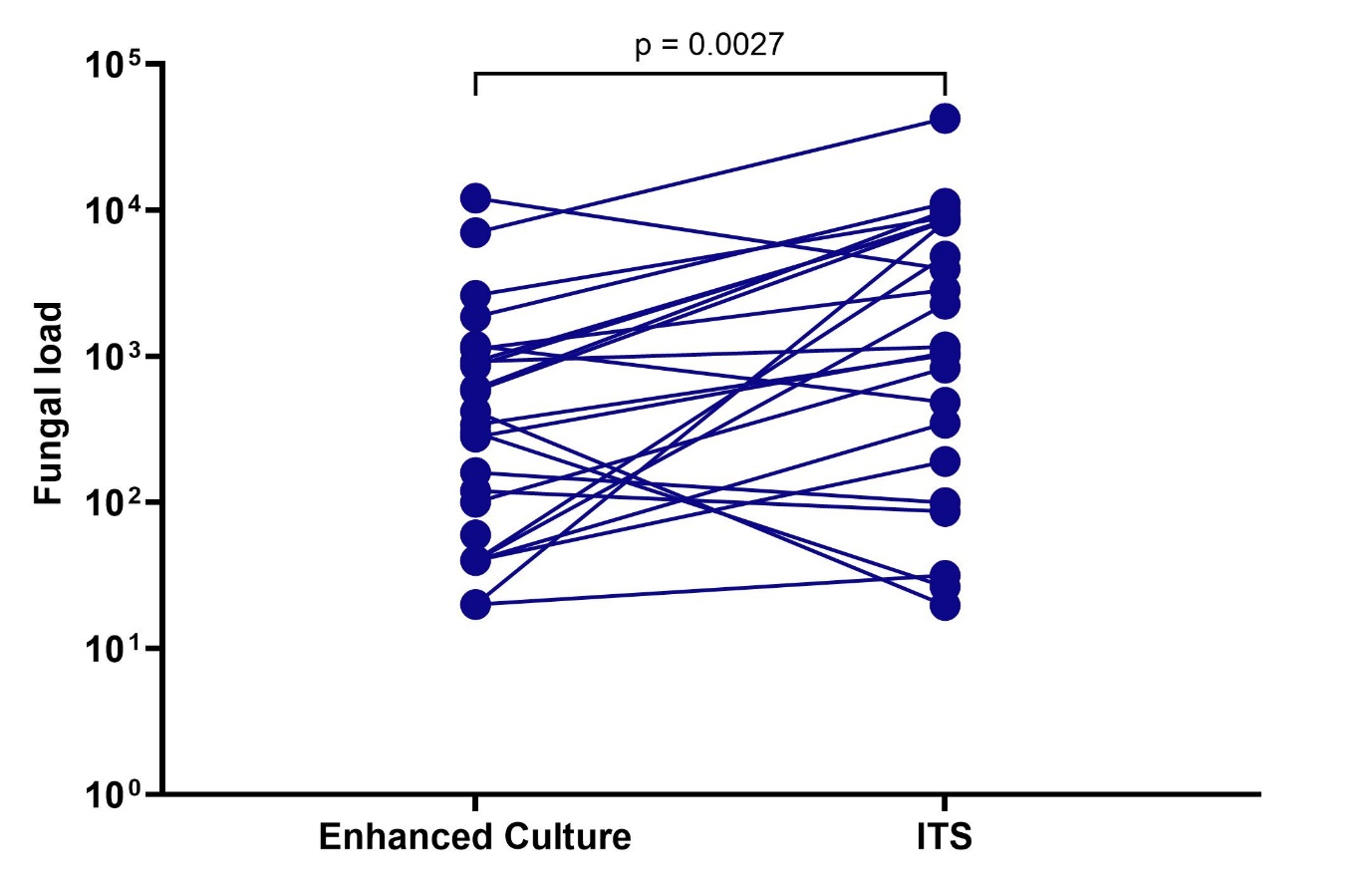


**Figure S2. Pairwise comparison of CFU/mL versus CFE/mL for fungal positive samples for enhanced culture and ITS qPCR.** Mann-Whitney test was performed on log transformed values. Significant differences at p<0.05.

**Table S1. Summary of fungal prevalence investigation results and patient clinical information.** Presence of fungi has been categorised as Fungal negative (FN) and Fungal positive (FP). Routine culture results provided by the diagnostic laboratories report for bacterial species have been categorised as Gram-positive, Gram-negative or Others. N/A – not available. NG- No colony growth in culture. RLI – Royal Lancaster Infirmary.

| Sample ID | Patient ID | Visit number | Grade | Stage | Routine culture | | | | Enhanced culture | | ITS qPCR | Location of swab | Outcome at 3 months |
| --- | --- | --- | --- | --- | --- | --- | --- | --- | --- | --- | --- | --- | --- |
|  |  |  |  |  | Fungi | Gram Positive | Gram Negative | Others | Colony isolation | MALDI-TOF |  |  |  |
| 1_2 | 1 | 2 | III | D | FN |  |  | Mixed skin | FN |  | FN | RLI | Still present but static |
| 1_3 |  | 3 | II | D | FN |  |  | Mixed skin | FN |  | FP | RLI | Still present but worsened |
| 2_2 | 2 | 2 | I | A | *C. albicans* |  | *Enterics* | Mixed skin | FP | *C. albicans* | FP | RLI | Still present but static |
| 2_3 |  | 3 | II | B | *C. albicans* | *S. aureus* | *Coliforms* |  | FP | *C. albicans* | FP | RLI | Still present but static |
| 2_4 |  | 4 | I | B | *C. albicans* | *S. aureus* | *Enterics* | Anaerobes | FP | *C. albicans* | FP | RLI | Still present but static |
| 2_5 |  | 5 | I | A | *C. albicans* | *S. aureus* | *Coliforms* |  | FP | *C. albicans* | FP | RLI | Still present but improved |
| 2_6 |  | 6 | I | A | *C. albicans* | *S. aureus* |  | Anaerobes | FN |  | FN | RLI | Still present but static |
| 2_7 |  | 7 | I | D | FN | *S. aureus* | *Enterics* |  | FP | *C. albicans* | FP | RLI | Still present but worsened |
| 2_8 |  | 8 | I | B | FN | *S. aureus* | *Mixed coliforms* | Anaerobes | FN |  | FP | RLI | Missing |
| 2_9 |  | 9 | I | B | FN | *S. aureus* | *Enterics* | Anaerobes, mixed skin | FN |  | FP | RLI | Missing |
| 3_2 | 3 | 2 | II | B | FN |  |  | Mixed skin | FN |  | FN | RLI | Still present but improved |
| 3_3 |  | 3 | I | A | FN |  | *Pseudomonas species* |  | FN |  | FN | Community | Missing |
| 4_1 | 4 | 1 | I | A | FN |  | *Enterics* |  | FN |  | FN | RLI | Healed |
| 5_1 | 5 | 1 | II | B | FN |  |  | NG | FN |  | FN | RLI | Still present but static |
| 5_2 |  | 2 | III | B | FN |  | *Mixed coliforms* |  | FN |  | FN | RLI | Still present but static |
| 5_3 |  | 3 | II | B | FN |  |  | Mixed skin | FN |  | FN | RLI | Still present but improved |
| 5_4 |  | 4 | III | B | FN | *C. striatum* |  | Mixed skin | FN |  | FN | RLI | Still present but improved |
| 5_5 |  | 5 | III | A | FN |  |  | Mixed skin | FN |  | FP | RLI | Still present but static |
| 5_6 |  | 6 | II | C | FN |  |  | Mixed skin | FN |  | FP | RLI | Amputated |
| 5_7 |  | 7 | III | B | FN |  |  | Mixed skin | FN |  | FN | RLI | Amputated |
| 5_8 |  | 8 | I | B | FN |  |  | Mixed skin | FN |  | FN | RLI | Healed |
| 5_9 |  | 9 | II | B | FN |  |  | Mixed skin, Anaerobes | FN |  | FN | RLI | Healed |
| 5_10 |  | 10 | I | A | FN |  |  | Mixed skin, Anaerobes | FN |  | FN | RLI | Still present but improved |
| 6_1 | 6 | 1 | I | B | FN | *S. aureus* | *Enterics* |  | FN |  | FN | RLI | Still present but worsened |
| 6_2 |  | 2 | I | A | FN |  |  | Mixed skin, Anaerobes | FN |  | FN | RLI | Still present but worsened |
| 6_3 |  | 3 | I | B | FN |  | *Pseudomonas species, Enterics* | Mixed skin | FN |  | FN | RLI | Still present but worsened |
| 7_1 | 7 | 1 | I | B | FN | *MRSA* |  |  | FN |  | FP | RLI | Healed |
| 8_1 | 8 | 1 | I | B | FN | *S. milleri* |  |  | FN |  | FN | RLI | Still present but worsened |
| 8_2 |  | 2 | III | D | FN |  | *P. aeruginosa* |  | FN |  | FN | RLI | Missing |
| 9_1 | 9 | 1 | I | B | FN |  |  | NG | FN |  | FN | RLI | Healed |
| 10_1 | 10 | 1 | II | B | *Candida sp* | *S. aureus* |  |  | FP | *C. parapsilosis^#^* | FP | RLI | Still present but static |
| 10_2 |  | 2 | I | D | *Candida sp* | *S. aureus* |  |  | FP | *C. parapsilosis^#^* | FN | RLI | Still present but worsened |
| 10_3 |  | 3 | II | B | FN |  | *Mixed coliforms* | Anaerobes | FN |  | FN | RLI | Still present but static |
| 10_4 |  | 4 | I | B | *Candida sp* |  | *Mixed coliforms* |  | FN |  | FN | RLI | Still present but improved |
| 10_5 |  | 5 | I | B | FN |  | *Coliforms* | Mixed skin | FN |  | FN | RLI | Still present but improved |
| 10_6 |  | 6 | II | B | FN |  | *Mixed coliforms* |  | FN |  | FP | RLI | Healed |
| 10_7_a |  | 7a | III | D | FN |  | *Coliforms bacilli* |  | FN |  | FN | RLI | Still present but improved |
| 10_7_b |  | 7b | I | D | FN |  | *Enterics* |  | FN |  | FP | RLI | Still present but improved |
| 10_8_a |  | 8a | II | D | FN |  | *Enterics* |  | FN |  | FP | RLI | Amputated |
| 10_8_b |  | 8b | I | B | FN |  | *Mixed coliforms* | Mixed skin | FN |  | FN | RLI | Healed |
| 10_9 |  | 9 | III | C | FN |  | *Mixed coliforms* |  | N/A |  | FN | RLI | Missing |
| 10_10_a |  | 10a | I | A | N/A | N/A | N/A | N/A | N/A |  | FN | RLI | Still present but improved |
| 10_10_b |  | 10b | II | B | N/A | N/A | N/A | N/A | N/A |  | FN | RLI | Still present but improved |
| 10_11_a |  | 11a | 0 | A | FN |  | *Enterics* |  | FP | *C. parapsilosis^#^* | FP | RLI | Missing |
| 10_11_b |  | 11b | I | B | *Candida sp* |  | *Mixed coliforms* | Mixed skin | FN |  | FP | RLI | Missing |
| 11_1 | 11 | 1 | I | B | FN |  |  | Mixed skin | FN |  | FN | RLI | Still present but worsened |
| 11_2 |  | 2 | I | B | FN |  |  | Mixed skin | FN |  | FN | RLI | Still present but improved |
| 11_3 |  | 3 | I | A | FN |  |  | Mixed skin | FN |  | FN | RLI | Still present but improved |
| 11_4 |  | 4 | I | A | FN |  |  | Mixed skin | FN |  | FP | RLI | Still present but improved |
| 12_1 | 12 | 1 | I | B | FN | *S. aureus* |  |  | FN |  | FP | RLI | Still present but improved |
| 13_1 | 13 | 1 | III | B | N/A | N/A | N/A | N/A | FN |  | FN | RLI | Still present but improved |
| 14_1 | 14 | 1 | I | B | FN |  |  | Mixed skin | FN |  | FN | RLI | Still present but improved |
| 15_1 | 15 | 1 | I | B | *C. albicans* |  | *Enterics, Pseudomonas species* |  | FN |  | FP | RLI | Healed |
| 15_2 |  | 2 | I | B | FN |  |  | NG | FN |  | FN | RLI | Healed |
| 16_1 | 16 | 1 | I | B | FN |  | *P. aeruginosa* | Anaerobes | FN |  | FP | RLI | Still present but static |
| 16_2 |  | 2 | I | B | FN |  | *Pseudomonas species* |  | FN |  | FN | RLI | Still present but improved |
| 16_3 |  | 3 | I | A | FN |  | *Coliforms* | Mixed skin | FN |  | FN | RLI | Still present but improved |
| 17_1 | 17 | 1 | II | B | FN |  | *Enterics, Pseudomonas species* |  | FN |  | FP | RLI | Still present but improved |
| 17_2 |  | 2 | II | B | *Candida sp* | *Enterococcus* | *Pseudomonas species* |  | FP | *C. glabrata* | FP | RLI | Healed |
| 17_3 |  | 3 | I | A | *Candida sp* |  | *Pseudomonas species* | Mixed skin | FN |  | FN | RLI | Still present but improved |
| 17_4 |  | 4 | III | B | FN |  |  | Mixed skin | FN |  | FN | RLI | Still present but improved |
| 18_1 | 18 | 1 | I | B | *C. albicans* | *Enterococcus* |  | Mixed skin | FN |  | FP | RLI | Still present but improved |
| 18_2 |  | 2 | I | A | *Candida sp* |  | *Mixed coliforms* | Mixed skin | FP | *C. glabrata* | FP | RLI | Still present but improved |
| 18_3 |  | 3 | I | A | FN | *Group B Strep* | *Coliforms* |  | FN |  | FN | RLI | Still present but improved |
| 18_4_a |  | 4a | II | B | FN | *S. aureus* |  |  | FN |  | FN | RLI | Healed |
| 18_4_b |  | 4b | II | B | *Candida sp* |  | *Enterics* |  | FP | *C. glabrata* | FP | RLI | Still present but improved |
| 18_5_a |  | 5a | I | B | FN | *S. aureus, Group G Strep* |  |  | FN |  | FN | RLI | Still present but improved |
| 18_5_b |  | 5b | I | B | FN | *S. aureus, Group G Strep* |  |  | FN |  | FN | RLI | Healed |
| 18_6 |  | 6 | I | B | FN | *S. aureus, Group G Strep* |  |  | FN |  | FN | RLI | Still present but improved |
| 18_7 |  | 7 | II | B | FN | *S. aureus* | *Enterics* |  | FN |  | FN | RLI | Missing |
| 19_1 | 19 | 1 | I | A | FN |  |  | Mixed skin | FN |  | FN | RLI | Amputated |
| 19_2 |  | 2 | III | B | FN |  |  | Mixed skin | FN |  | FP | RLI | Still present but improved |
| 19_3 |  | 3 | III | B | FN |  |  | Mixed skin | FN |  | FN | RLI | Still present but improved |
| 20_1 | 20 | 1 | I | B | FN | *S. aureus, Enterococcus* |  |  | FN |  | FN | RLI | Still present but worsened |
| 20_2 |  | 2 | I | B | FN | *S. aureus* |  |  | FN |  | FN | RLI | Still present but static |
| 20_3 |  | 3 | III | B | FN | *S. aureus* | *Enterics* |  | FN |  | FN | RLI | Amputated |
| 20_4_a |  | 4a | I | A | FN | *S. aureus* | *Enterics* |  | FN |  | FN | RLI | Missing |
| 20_4_b |  | 4b | I | A | FN | *S. aureus* | *Enterics* |  | FN |  | FN | RLI | Missing |
| 21_1 | 21 | 1 | I | B | FN | *S. aureus* |  |  | FN |  | FN | RLI | Still present but improved |
| 22_1 | 22 | 1 | I | A | FN | *E. faecalis* | *P. aeruginosa* | Mixed skin | FN |  | FN | RLI | Still present but improved |
| 22_2 |  | 2 | II | B | FN |  | *Enterics, Pseudomonas species* |  | FN |  | FN | RLI | Still present but improved |
| 22_3 |  | 3 | I | A | FN |  |  | Mixed skin | FP | *R. mucilaginosa* | FP | RLI | Still present but improved |
| 22_4 |  | 4 | I | A | FN |  |  | Mixed skin | FN |  | FN | RLI | Still present but static |
| 22_5 |  | 5 | III | A | FN |  |  | Mixed skin | FN |  | FN | RLI | Still present but improved |
| 22_6_a |  | 6a | III | B | FN |  | *Enterics* |  | FN |  | FP | RLI | Still present but improved |
| 22_6_b |  | 6b | I | A | FN |  | *Coliforms bacilli* | Mixed skin | N/A | N/A | FN | RLI | Still present but improved |
| 23_1 | 23 | 1 | II | B | FN | *Group B Strep* |  |  | FN |  | FN | RLI | Still present but worsened |
| 23_2 |  | 2 | I | A | FN |  | *Enterics* |  | FN |  | FN | RLI | Still present but improved |
| 23_3 |  | 3 | I | A | *Candida sp* |  | *Enterics* |  | FN |  | FN | RLI | Missing |
| 24_1 | 24 | 1 | I | C | FN |  |  | NG | FN |  | FN | RLI | Healed |
| 24_2 |  | 2 | I | C | *Candida sp* |  |  | Mixed skin | FP | *C. parapsilosis*^†^ | FP | RLI | Amputated |
| 24_3 |  | 3 | III | D | FN |  |  | Mixed skin | FN |  | FN | RLI | Amputated |
| 24_4 |  | 4 | I | C | *Candida sp* | *S. aureus* | *Mixed coliforms* |  | FP | *C. parapsilosis*^†^ | FP | RLI | Amputated |
| 25_1 | 25 | 1 | III | B | FN |  |  | Mixed skin | FN |  | FN | RLI | Healed |
| 26_1 | 26 | 1 | I | A | FN |  | *Enterics* | Mixed skin | FN |  | FN | RLI | Missing |
| 27_1 | 27 | 1 | II | B | FN |  |  | Mixed skin | FN |  | FN | RLI | Still present but improved |
| 27_2 |  | 2 | II | B | FN |  |  | Mixed skin | FN |  | FP | RLI | Still present but improved |
| 27_3 |  | 3 | I | B | FN | *S. aureus* | *Enterics* |  | FN |  | FP | RLI | Still present but improved |
| 27_4 |  | 4 | II | B | FN | *S. aureus* | *Enterics* |  | FN |  | FP | RLI | Healed |
| 27_5 |  | 5 | III | D | FN |  | *Enterics, Pseudomonas species* |  | FN |  | FP | RLI | Missing |
| 27_6 |  | 6 | II | B | FN |  | *Enterics* |  | FN |  | FP | RLI | Missing |
| 28_1 | 28 | 1 | I | B | FN |  | *Enterics, Pseudomonas species* | Mixed skin | FN |  | FN | RLI | Still present but static |
| 28_2 |  | 2 | I | A | FN | *MRSA* |  |  | FN |  | FN | RLI | Still present but static |
| 28_3 |  | 3 | I | A | FN |  | *Pseudomonas oleovorans* |  | FN |  | FN | RLI | Still present but improved |
| 28_4 |  | 4 | I | C | FN | *MRSA* | *Enterics* |  | FP | *C. parapsilosis*^†^ | FN | RLI | Still present but improved |
| 28_5 |  | 5 | I | B | FN |  | *Enterics* |  | FN |  | FN | RLI | Still present but static |
| 28_6 |  | 6 | II | A | FN | *MRSA* | *Enterics* |  | FN |  | FN | RLI | Still present but static |
| 28_7 |  | 7 | I | C | FN | *MRSA* |  |  | FN |  | FN | RLI | Missing |
| 28_8 |  | 8 | I | A | FN |  | *Enterics* |  | FN |  | FN | RLI | Missing |
| 29_1 | 29 | 1 | I | A | FN | *MRSA* |  |  | FN |  | FN | RLI | Healed |
| 30_1 | 30 | 1 | 0 | A | N/A | N/A | N/A | N/A | FN |  | FN | RLI | Healed |
| 31_1 | 31 | 1 | III | B | FN |  |  | NG | FN |  | FN | RLI | Still present but improved |
| 32_1 | 32 | 1 | I | A | FN |  |  | Mixed skin | FN |  | FN | RLI | Still present but improved |
| 33_1 | 33 | 1 | III | B | FN |  |  | Mixed skin | FN |  | FN | RLI | Still present but improved |
| 33_2 |  | 2 | III | B | FN | *C. striatum* |  | Mixed skin | FN |  | FN | RLI | Healed |
| 34_1 | 34 | 1 | I | B | FN | *S. aureus* | *Mixed coliforms* | Anaerobes | FN |  | FP | RLI | Still present but improved |
| 34_2 |  | 2 | I | B | FN |  | *Mixed coliforms, Pseudomonas species* |  |  |  | FN | RLI | Healed |
| 34_3 |  | 3 | I | B | FN |  | *Coliforms* | Mixed skin | FN |  | FN | RLI | Healed |
| 35_1 | 35 | 1 | I | B | FN |  |  | NG | FN |  | FN | RLI | Healed |
| 36_1 | 36 | 1 | III | B | FN | *Group B Strep* |  |  | FN |  | FN | RLI | Still present but improved |
| 37_1 | 37 | 1 | I | B | FN |  |  | Mixed skin | FN |  | FN | RLI | Still present but improved |
| 37_2 |  | 2 | I | B | FN |  |  | Mixed skin | FN |  | FN | RLI | Still present but improved |
| 38_1 | 38 | 1 | III | D | FN | *S. aureus* | *Mixed coliforms, Pseudomonas species* |  | FN |  | FN | RLI | Still present but improved |
| 38_2 |  | 2 | I | B | FN | *S. aureus* | *Enterics* |  | FN |  | FN | RLI | Still present but improved |
| 38_3 |  | 3 | III | D | FN |  | *Enterics, Pseudomonas species* |  | FN |  | FN | RLI | Still present but improved |
| 38_4 |  | 4 | I | A | FN | *S. simulans* | *E. cloacae* |  | FN |  | FN | RLI | Still present but improved |
| 38_5 |  | 5 | II | B | FN |  |  | Mixed skin | FN |  | FN | RLI | Still present but static |
| 38_6 |  | 6 | I | B | FN |  |  | Mixed skin | FN |  | FP | RLI | Healed |
| 38_7 |  | 7 | I | A | FN |  |  | Mixed skin | FN |  | FN | RLI | Still present but improved |
| 38_8 |  | 8 | III | B | FN |  |  | Mixed skin, Anaerobes | FN |  | FN | RLI | Healed |
| 38_9 |  | 9 | III | B | FN |  |  | Mixed skin |  |  | FN | RLI | Still present but worsened |
| 38_10 |  | 10 | III | B | FN |  | *Pseudomonas species* |  | FN |  | FN | RLI | Missing |
| 39_1 | 39 | 1 | I | B | FN | *S. aureus* | *Mixed coliforms* |  | FN |  | FP | RLI | Healed |
| 40_1 | 40 | 1 | III | B | FN |  | *Mixed coliforms* |  | FN |  | FN | RLI | Still present but improved |
| 41_1 | 41 | 1 | I | A | FN | *S. aureus* |  |  | FN |  | FN | Community | Still present but static |
| 41_2 |  | 2 | I | A | FN | *S. aureus* |  | Anaerobes | FN |  | FN | Community | Still present but static |
| 42_1 | 42 | 1 | II | A | FN |  |  | NG | FN |  | FN | Community | Still present but improved |
| 42_2 |  | 2 | I | A | FN |  |  | NG | FN |  | FN | Community | Missing |
| 43_1 | 43 | 1 | I | C | N/A | N/A | N/A | N/A | FN |  | FN | Community | Healed |
| 44_1 | 44 | 1 | II | B | FN |  | *Proteus species* |  | FN |  | FN | Community | Healed |
| 45_1 | 45 | 1 | I | C | FN |  | *Mixed coliforms* |  | FN |  | FP | Community | Still present but static |
| 46_1 | 46 | 1 | I | A | FN |  | *Mixed coliforms, Pseudomonas species* |  | FN |  | FN | Community | Still present but improved |
| 46_2 |  | 2 | I | A | FN |  | *Mixed coliforms, Pseudomonas species* |  | FN |  | FP | Community | Still present but improved |
| 46_3 |  | 3 | I | A | FN |  | *Coliform, Pseudomonas species* |  | FN |  | FN | Community | Still present but improved |
| 47_1 | 47 | 1 | I | B | FN |  |  | Mixed skin | FN |  | FP | RLI | Still present but worsened |
| 47_2 |  | 2 | I | B | FN |  |  | NG | FN |  | FN | RLI | Still present but static |
| 47_3 |  | 3 | I | A | FN |  |  | FN | FN |  | FN | RLI | Still present but improved |
| 47_4 |  | 4 | III | D | FN |  |  | Mixed skin | FN |  | FN | RLI | Still present but improved |
| 47_5 |  | 5 | I | B | N/A | N/A | N/A | N/A | FN |  | FN | RLI | Still present but improved |
| 47_6 |  | 6 | I | C | FN |  |  | Mixed skin, Anaerobes | FN |  | FN | RLI | Still present but worsened |
| 47_7_a |  | 7a | I | B | FN |  |  | Mixed skin, Anaerobes | FN |  | FN | RLI | Missing |
| 47_7_b |  | 7b | I | B | FN |  |  | Mixed skin, Anaerobes | FN |  | FN | RLI | Missing |
| 48_1 | 48 | 1 | I | B | FN | *S. aureus* |  |  | FN |  | FN | RLI | Still present but improved |
| 48_2 |  | 2 | II | B | FN | *Group B Strep* |  |  | FN |  | FN | RLI | Healed |
| 48_3 |  | 3 | II | B | FN |  |  | Mixed skin | FN |  | FN | RLI | Healed |
| 49_1 | 49 | 1 | I | A | FN |  |  | No signifcant growth | FN |  | FN | Community | Still present but static |
| 50_1 | 50 | 1 | I | A | FN |  |  | No signifcant growth | FN |  | FN | Community | Healed |
| 51_1 | 51 | 1 | I | A | FN |  |  | No signifcant growth | FN |  | FN | Community | Healed |
| 52_1 | 52 | 1 | I | A | FN |  | *Enterics* |  | FN |  | FN | Community | Healed |
| 53_1 | 53 | 1 | I | A | FN |  |  | No signifcant growth | FN |  | FN | Community | Still present but improved |
| 53_2 |  | 2 | I | A | FN |  |  | No signifcant growth | FN |  | FN | Community | Still present but improved |
| 54_1 | 54 | 1 | I | A | FN |  |  | NG | FN |  | FP | Community | Still present but improved |
| 54_2 |  | 2 | I | A | FN | *S. aureus* |  |  | FN |  | FN | Community | Still present but improved |
| 55_1 | 55 | 1 | I | C | FN | *S. aureus* | *Coliforms* |  | FN |  | FN | Community | Still present but static |
| 55_2 |  | 2 | III | A | FN |  |  | NG | FN |  | FN | Community | Still present but improved |
| 56_1 | 56 | 1 | I | A | FN | *S. aureus* | *coliforms* |  | FN |  | FN | Community | Still present but worsened |
| 56_2 |  | 2 | I | A | FN | *S. aureus* |  |  | FN |  | FP | Community | Still present but worsened |
| 56_3 |  | 3 | II | B | FN | *S. aureus* | *Proteus species* |  | FN |  | FN | Community | Still present but worsened |
| 57_1 | 57 | 1 | I | B | FN |  | *Enterics* |  | FN |  | FP | RLI | Still present but improved |
| 57_2 |  | 2 | I | B | FN | *Group C Strep* | *Enterics* |  | FN |  | FP | RLI | Still present but improved |
| 57_3 |  | 3 | I | A | FN |  | *Enterics* | Mixed skin | FN |  | FP | RLI | Still present but improved |
| 57_4 |  | 4 | I | B | FN |  | *Mixed enteric* |  | FN |  | FN | RLI | Still present but improved |
| 57_5 |  | 5 | I | A | FN | *Group G Strep* |  | Mixed skin | FN |  | FP | RLI | Still present but worsened |
| 57_6 |  | 6 | I | B | FN |  | *Enterics, Pseudomonas species* |  | FN |  | FN | RLI | Healed |
| 57_7_a |  | 7a | I | B | *C. albicans* | *MRSA* | *Enterics* |  | FN |  | FP | RLI | Healed |
| 57_7_b |  | 7b | I | A | FN | *MRSA* |  |  | FP | *C. albicans* | FP | RLI | Healed |
| 57_8 |  | 8 | I | B | FN | *MRSA* |  |  | FN |  | FP | RLI | Still present but static |
| 57_9 |  | 9 | II | B | FN |  | *Enterics* |  | FN |  | FN | RLI | Missing |
| 57_10 |  | 10 | III | B | N/A | N/A | N/A | N/A | FN |  | FN | RLI | Still present but worsened |
| 57_11 |  | 11 | III | B | FN |  | *Enterics* |  | FN |  | FP | RLI | Still present but worsened |
| 58_1 | 58 | 1 | I | A | FN |  | *Coliforms* | Mixed skin | N/A |  | FN | RLI | Healed |
| 59_1 | 59 | 1 | I | A | FN |  | *Coliforms* |  | FN |  | FN | Community | Still present but worsened |
| 60_1 | 60 | 1 | I | A | FN | *S. aureus* |  |  | FN |  | FN | Community | Still present but improved |
| 60_2 |  | 2 | II | A | FN |  | *Mixed coliforms* |  | FN |  | FP | Community | Still present but improved |
| 61_1 | 61 | 1 | I | B | FN |  | *Coliforms* |  | FN |  | FN | Community | Still present but improved |
| 62_1 | 62 | 1 | 0 | A | FN | *S. aureus, Group B Strep* |  |  | FN |  | FN | Community | Still present but static |
| 63_1 | 63 | 1 | I | B | FN | *S. aureus* | *Enterics* |  | FN |  | FN | Community | Still present but improved |
| 64_1 | 64 | 1 | II | B | N/A |  |  |  | FN |  | FN | RLI | Still present but improved |
| 64_2 |  | 2 | III | A | N/A | N/A | N/A | N/A | FN |  | FP | RLI | Still present but improved |
| 65_1 | 65 | 1 | I | A | FN | *S. aureus* | *Coliforms* |  | FN |  | FP | RLI | Still present but improved |
| 65_2 |  | 2 | I | A | FN | *S. aureus* |  |  | FN |  | FP | RLI | Still present but improved |
| 65_3 |  | 3 | I | D | N/A |  |  |  | FN |  | FN | RLI | Still present but worsened |
| 65_4 |  | 4 | I | A | FN | *S. aureus* | *Mixed coliforms* |  | FN |  | FN | RLI | Still present but static |
| 65_5 |  | 5 | III | D | FN |  | *Mixed coliforms* | Mixed skin | FN |  | FN | RLI | Missing |
| 65_6 |  | 6 | I | A | FN | *S. aureus* | *Mixed coliforms* |  | FN |  | FN | RLI | Still present but improved |
| 65_7 |  | 7 | I | A | FN |  |  | Mixed skin | FN |  | FP | RLI | Missing |
| 65_8 |  | 8 | I | A | FN | *S. aureus* |  |  | FN |  | FP | RLI | Missing |
| 66_1 | 66 | 1 | II | A | FN |  | *Mixed coliforms, Pseudomonas species* |  | FN |  | FN | RLI | Still present but improved |
| 66_2 |  | 2 | II | B | FN |  | *Mixed coliforms, Pseudomonas species* |  | FN |  | FN | RLI | Healed |
| 66_3 |  | 3 | II | B | FN |  | *Coliforms* | Mixed skin | FN |  | FN | RLI | Healed |
| 67_1 | 67 | 1 | II | B | FN | *S. aureus* |  |  | FN |  | FP | RLI | Missing |
| 68_1 | 68 | 1 | I | A | FN | *S. aureus* |  |  | FN |  | FN | RLI | Still present but improved |
| 68_2 |  | 2 | II | B | FN | *S. aureus* |  |  | FN |  | FP | RLI | Still present but improved |
| 68_3 |  | 3 | I | D | FN | *S. aureus* | *Coliforms bacilli* |  | FN |  | FN | RLI | Still present but worsened |
| 68_4_a |  | 4a | I | B | FN |  | *Enterics* | Mixed skin | FN |  | FN | RLI | Still present but static |
| 68_4_b |  | 4b | I | B | FN |  | *Enterics* | Mixed skin | FN |  | FN | RLI | Still present but static |
| 69_1 | 69 | 1 | III | D | FN |  | *Enterics* | Anaerobes | FN |  | FN | RLI | Patient Deceased |
| 70_1 | 70 | 1 | I | A | FN | *S. aureus* |  |  | FN |  | FP | Community | Still present but improved |
| 71_1 | 71 | 1 | I | A | FN |  |  | NG | FP | *C. parapsilosis ^#,^* ^†^ | FP | Community | Healed |
| 72_1 | 72 | 1 | I | B | FN |  | *Coliforms* | Mixed skin | FN |  | FN | RLI | Still present but static |
| 72_2 |  | 2 | I | A | FN |  |  | NG | FN |  | FN | RLI | Still present but worsened |
| 73_1 | 73 | 1 | I | A | FN | *S. lugdunensis* |  |  | FN |  | FP | Community | Still present but improved |
| 74_1 | 74 | 1 | II | A | FN |  | *Coliforms bacilli* |  | FN |  | FP | Community | Still present but improved |
| 75_1 | 75 | 1 | I | B | FN | *S. aureus* |  |  | FN |  | FP | Community | Healed |
| 76_1 | 76 | 1 | I | B | FN |  | *Enterics* |  | FN |  | FP | RLI | Still present but improved |
| 76_2 |  | 2 | II | B | FN |  |  | Mixed skin | FN |  | FP | RLI | Still present but improved |
| 77_1 | 77 | 1 | I | A | FN |  |  | NG | FN |  | FP | Community | Healed |
| 78_1 | 78 | 1 | I | A | FN | *S. aureus* |  |  | FN |  | FN | Community | Still present but static |
| 79_1 | 79 | 1 | I | A | FN | *S. aureus* |  |  | FN |  | FP | Community | Healed |
| 80_1 | 80 | 1 | II | B | FN |  | *Coliforms* | Mixed skin | FN |  | FN | RLI | Still present but improved |
| 80_2 |  | 2 | II | B | FN |  |  | NG | FN |  | FN | RLI | Patient Deceased |
| 80_3 |  | 3 | I | A | FN |  | *Coliforms* | Mixed skin | FN |  | FP | RLI | Patient Deceased |
| 80_4 |  | 4 | I | B | FN |  |  | Mixed skin | FN |  | FN | RLI | Patient Deceased |
| 81_1 | 81 | 1 | I | A | FN |  |  | Mixed skin | FN |  | FN | RLI | Still present but worsened |
| 82_1 | 82 | 1 | I | A | FN |  |  | Anaerobes | FN |  | FN | Community | Still present but improved |
| 83_1 | 83 | 1 | I | A | FN |  |  | NG | FN |  | FN | Community | Healed |
| 84_1 | 84 | 1 | I | A | FN |  | *Mixed coliforms* |  | FN |  | FP | RLI | Healed |
| 85_1 | 85 | 1 | III | B | FN |  | *Enterics, Pseudomonas species* |  | FN |  | FN | RLI | Still present but worsened |
| 85_2 |  | 2 | II | B | FN |  |  | FN | FN |  | FN | RLI | Still present but static |
| 85_3 |  | 3 | III | A | FN |  | *Enterics, Pseudomonas species* |  | FP | *C. parapsilosis^#^* | FN | RLI | Still present but improved |
| 85_4 |  | 4 | III | B | FN |  | *P. aeruginosa* | Mixed skin | FN |  | FN | RLI | Still present but improved |
| 85_5 |  | 5 | I | A | FN |  |  | Mixed skin | FN |  | FP | RLI | Still present but improved |
| 85_6 |  | 6 | I | A | FN |  |  | Mixed skin | FN |  | FN | RLI | Still present but improved |
| 86_1 | 86 | 1 | III | B | FN | *S. aureus* |  |  | FN |  | FP | RLI | Healed |
| 87_1 | 87 | 1 | III | B | FN |  | *Mixed coliforms* | Anaerobes | FN |  | FN | Community | Healed |
| 88_1 | 88 | 1 | I | A | FN | *S. aureus* |  |  | FN |  | FP | Community | Healed |
| 88_2 |  | 2 | I | D | FN | *S. aureus* |  |  | N/A |  | FN | Community | Healed |
| 89_1 | 89 | 1 | I | A | FN |  | *Coliforms* | Mixed skin, Anaerobes | FN |  | FP | Community | Still present but improved |
| 90_1 | 90 | 1 | III | D | FN | *S. aureus* |  |  | FN |  | FP | Community | Still present but improved |
| 91_1 | 91 | 1 | III | B | FN |  | *Coliforms* | Mixed skin | FN |  | FN | RLI | Still present but improved |
| 91_2 |  | 2 | III | B | FN |  |  | No signifcant growth | FN |  | FP | RLI | Healed |
| 91_3 |  | 3 | I | B | FN |  | *Mixed coliforms, Pseudomonas species* | Mixed skin | FN |  | FN | RLI | Still present but improved |
| 92_1 | 92 | 1 | III | D | FN |  |  | Mixed skin | FN |  | FN | RLI | Still present but improved |
| 92_2 |  | 2 | III | B | FN |  | *Enterics* |  | FN |  | FP | RLI | Still present but improved |
| 92_3 |  | 3 | III | B | FN |  |  | Mixed skin | FN |  | FN | RLI | Healed |
| 93_1 | 93 | 1 | III | B | FN | *S. aureus, Group B Strep* |  |  | FN |  | FP | RLI | Still present but improved |
| 93_2 |  | 2 | I | B | FN | *Group B Strep* |  |  | FN |  | FN | RLI | Still present but static |
| 93_3 |  | 3 | I | B | FN | *S. aureus, Group B Strep* |  |  | FN |  | FP | RLI | Missing |
| 93_4 |  | 4 | I | B | FN | *Group B Strep* |  | Mixed skin | FN |  | FP | RLI | Still present but worsened |
| 93_5 |  | 5 | I | B | FN | *S. aureus, Group B Strep* |  |  | FN |  | FN | RLI | Still present but improved |
| 93_6 |  | 6 | I | B | FN | *S. aureus, Group B Strep* |  |  | FN |  | FP | RLI | Still present but improved |
| 93_7 |  | 7 | III | B | FN | *S. aureus* | *Enterics* |  | FN |  | FP | RLI | Still present but improved |
| 93_8 |  | 8 | III | D | FN |  | *Coliforms bacilli* | Mixed skin | FN |  | FP | RLI | Healed |
| 93_9 |  | 9 | III | B | FN | *S. aureus* | *Enterics* |  | FN |  | FN | RLI | Still present but static |
| 93_10 |  | 10 | III | B | FN | *S. aureus* | *Enterics* |  | FN |  | FN | RLI | Still present but static |
| 93_11 |  | 11 | III | B | FN |  |  | Mixed skin | FN |  | FN | RLI | Amputated |
| 93_12 |  | 12 | III | B | FN |  |  | Mixed skin | FN |  | FP | RLI | Amputated |
| 93_13 |  | 13 | I | B | FN |  |  | Mixed skin | FN |  | FP | RLI | Amputated |
| 94_1 | 94 | 1 | I | B | FN |  | *Enterics* |  | FN |  | FN | RLI | Still present but improved |
| 95_1 | 95 | 1 | III | A | FN |  | *Enterics* |  | FN |  | FN | RLI | Still present but improved |
| 95_2 |  | 2 | III | B | FN |  |  | Mixed skin, Anaerobes | FN |  | FP | RLI | Still present but improved |
| 95_3 |  | 3 | III | B | FN |  |  | Mixed skin | FN |  | FN | RLI | Still present but static |
| 95_4 |  | 4 | II | B | FN |  |  | Anaerobes, mixed skin | FN |  | FN | RLI | Missing |
| 95_5 |  | 5 | III | B | FN |  | *Coliforms bacilli* | Mixed skin | FN |  | FN | RLI | Missing |
| 95_6 |  | 6 | III | C | N/A | N/A | N/A | N/A | FN |  | FN | RLI | Missing |
| 95_7 |  | 7 | III | D | N/A | N/A | N/A | N/A | FN |  | FN | RLI | Missing |
| 96_1 | 96 | 1 | I | D | FN |  | *Enterics* |  | FN |  | FN | RLI | Missing |
| 97_1 | 97 | 1 | III | B | FN |  |  | Anaerobes | FN |  | FN | RLI | Still present but improved |
| 97_2 |  | 2 | III | D | FN |  |  | Mixed skin, Anaerobes | FN |  | FP | RLI | Amputated |
| 97_3 |  | 3 | III | B | FN |  |  | Mixed skin, Anaerobes | FN |  | FP | RLI | Amputated |
| 97_4 |  | 4 | I | B | FN |  | *Coliforms bacilli* | mixed skin | FN |  | FP | RLI | Healed |
| 97_5 |  | 5 | II | A | FN |  | *Coliforms bacilli* |  | N/A |  | FN | RLI | Healed |
| 98_1 | 98 | 1 | I | A | FN |  |  | Mixed skin | FN |  | FN | RLI | Healed |
| 99_1 | 99 | 1 | III | D | FN | *S. aureus* | *Enterics* |  | FN |  | FN | RLI | Missing |
| 99_2 |  | 2 | III | B | FN |  | *Enterics* |  | FN |  | FN | RLI | Missing |
| 99_3 |  | 3 | III | B | FN | *S. aureus* | *Mixed coliforms* |  | FN |  | FN | RLI | Still present but improved |
| 99_4 |  | 4 | III | A | FN | *Group G Strep* | *Mixed coliforms, Pseudomonas species* |  | FN |  | FN | RLI | Missing |
| 99_5 |  | 5 | I | D | FN | *Group G Strep,*  *S. aureus* | *Mixed coliforms, Pseudomonas species* |  | FN |  | FN | RLI | Missing |
| 99_6 |  | 6 | III | B | FN |  |  | FN | FN |  | FN | RLI | Missing |
| 100_1_a | 100 | 1a | II | B | FN |  | *Enterics* | Mixed skin | FN |  | FN | RLI | Still present but improved |
| 100_1_b |  | 1b | II | B | FN |  | *coliforms bacilli* |  | FN |  | FN | RLI | Still present but improved |
| 101_1 | 101 | 1 | II | B | *Candida sp* |  | *Enterics* | Mixed skin | FP | *C. parapsilosis^#^* | FP | RLI | Healed |
| 102_1 | 102 | 1 | I | A | FN |  |  | Mixed skin | FP | *C. parapsilosis^#^* | FP | RLI | Healed |
| 102_2 |  | 2 | I | B | FN |  | *Enterics, Pseudomonas species* |  | FN |  | FN | RLI | Still present but improved |
| 102_3 |  | 3 | I | B | *Candida sp* |  | *Enterics* |  | FP | *C. parapsilosis*^†^ | FN | RLI | Still present but improved |
| 102_4 |  | 4 | I | A | *Candida sp* |  | *Enterics* |  | FP | *C. parapsilosis^#^* | FP | RLI | Missing |
| 103_1 | 103 | 1 | III | D | FN | *S. aureus* |  |  | FP | *C. parapsilosis^#^* | FN | RLI | Missing |
| 104_1 | 104 | 1 | I | A | FN |  |  | Mixed skin | FN |  | FN | Community | Missing |
| 105_1 | 105 | 1 | I | A | *C. albicans Candida sp* |  | *Enterics* |  | FN |  | FN | Community | Still present but improved |
| 106_1 | 106 | 1 | I | B | FN |  |  | mixed skin | FN |  | FN | RLI | Healed |
| 107_1 | 107 | 1 | I | A | FN |  |  | NG | FN |  | FP | RLI | Healed |
| 108_1 | 108 | 1 | I | B | FN |  | *Enterics* |  | FN |  | FN | Community | Healed |
| 109_1 | 109 | 1 | III | B | FN |  |  | *Mixed skin* | FN |  | FP | RLI | Still present but worsened |
| 109_2 |  | 2 | I | B | FN | *Enterococcus species* |  | *Mixed skin* | FN |  | FN | RLI | Missing |
| 109_3 |  | 3 | III | B | N/A | N/A | N/A | N/A | FN |  | FN | RLI | Missing |
| 109_4 |  | 4 | I | B | FN |  |  | Mixed skin | FN |  | FN | RLI | Amputated |
| 110_1 | 110 | 1 | I | B | FN |  |  | Mixed skin | FN |  | FN | RLI | Still present but static |
| 111_1 | 111 | 1 | II | B | FN |  |  | Mixed skin | FN |  | FN | RLI | Still present but improved |
| 111_2 |  | 2 | II | C | FN | *Group B Strep* |  | Mixed skin | FN |  | FN | RLI | Still present but static |
| 111_3 |  | 3 | III | C | *Candida sp* |  |  | Anaerobes | FP | *C. parapsilosis^#^* | FP | RLI | Missing |
| 111_4 |  | 4 | III | C | *Candida sp* |  |  | Anaerobes, mixed skin | FP | *C. parapsilosis^#^* | FP | RLI | Missing |
| 111_5 |  | 5 | III | C | FN | *S. aureus* |  |  | FN |  | FN | RLI | Missing |
| 111_6 |  | 6 | III | D | *Candida sp* | *S. aureus* |  | Anaerobes | FP | *C. parapsilosis^#^* | FP | RLI | Missing |
| 112_1 | 112 | 1 | I | B | FN |  | *Enterics, Pseudomonas species* |  | FN |  | FN | RLI | Still present but worsened |
| 112_2_a |  | 2a | I | B | *Candida sp* |  | *Pseudomonas species* |  | FP | *C. parapsilosis^#^* | FP | RLI | Healed |
| 112_2_b |  | 2b | I | B | FN |  | *Pseudomonas species* | Anaerobes | FN |  | FP | RLI | Healed |
| 112_3 |  | 3 | I | B | FN |  | *Mixed coliforms, Pseudomonas species* | Anaerobes | FP | *C. parapsilosis^#^* | FP | RLI | Healed |
| 113_1 | 113 | 1 | III | B | FN |  | *Mixed coliforms* |  | FN |  | FN | RLI | Still present but improved |
| 113_2 |  | 2 | III | B | FN |  |  | Mixed skin | FN |  | FN | RLI | Healed |
| 113_3 |  | 3 | I | B | FN |  |  | Mixed skin | FN |  | FN | RLI | Healed |
| 114_1 | 114 | 1 | I | A | FN |  |  | NG | FN |  | FN | RLI | Healed |
| 114_2 |  | 1 | I | A | FN |  |  | FN | FN |  | FN | RLI | Healed |
| 115_1 | 115 | 1 | I | B | FN |  | *Mixed coliforms* |  | FN |  | FN | RLI | Healed |
| 116_1 | 116 | 1 | I | B | FN |  | *P. aeruginosa* |  | FN |  | FN | RLI | Healed |
| 116_2 |  | 2 | III | B | FN | *S. aureus* | *Enterics* |  | FN |  | FN | RLI | Missing |
| 116_3 |  | 3 | I | B | FN |  | *Enterics, Pseudomonas species* |  | FN |  | FN | RLI | Missing |
| 116_4 |  | 4 | II | B | FN |  | *Enterics, Pseudomonas species* |  | FN |  | FP | RLI | Missing |
| 117_1 | 117 | 1 | III | D | FN |  | *Mixed coliforms* |  | FN |  | FN | RLI | Still present but improved |
| 117_2 |  | 2 | III | B | FN |  | *Mixed coliforms, Pseudomonas species* |  | FN |  | FN | RLI | Still present but improved |
| 117_3 |  | 3 | III | D | FN |  | *Enterics* |  | FN |  | FN | RLI | Still present but improved |
| 117_4_a |  | 4a | I | B | FN |  | *Enterics, Pseudomonas species* |  | FN |  | FN | RLI | Missing |
| 117_4_b |  | 4b | III | B | FN |  | *Enterics, Pseudomonas species* | mixed skin | FN |  | FN | RLI | Missing |
| 117_5_a |  | 5a | I | B | FN |  | *Enterics* |  | FN |  | FP | RLI | Missing |
| 117_5_b |  | 5b | III | B | FN |  | *Enterics, Pseudomonas species* |  | FN |  | FN | RLI | Missing |
| 117_6 |  | 6 | I | B | FN |  | *Enterics, Pseudomonas species* |  | FN |  | FP | RLI | Healed |
| 118_1 | 118 | 1 | III | B | FN |  | *Enterics* | Anaerobes | FN |  | FN | RLI | Still present but improved |
| 118_2 |  | 2 | III | B | FN |  | *Enterics* | Mixed skin | FN |  | FN | RLI | Healed |
| 118_3 |  | 3 | I | A | FN |  |  | mixed skin | FN |  | FN | RLI | Missing |
| 119_1 | 119 | 1 | III | D | FN |  |  | Mixed skin | FN |  | FN | RLI | Still present but improved |
| 119_2 |  | 2 | II | A | FN |  |  | Mixed skin | FN |  | FN | RLI | Still present but worsened |
| 120_1 | 120 | 1 | III | B | *C. albicans* |  | *Enterics* |  | FP | *C. albicans* | FN | RLI | Missing |
| 122_1 | 122 | 1 | III | B | FN |  |  | Anaerobes, mixed skin | FN |  | FP | RLI | Still present but improved |
| 122_2 |  | 2 | I | A | FN |  | *Mixed coliforms* | Anaerobes | FN |  | FN | RLI | Still present but improved |
| 122_3_a |  | 3a | I | B | FN |  |  | Mixed skin | FN |  | FP | RLI | Missing |
| 122_3_b |  | 3b | I | A | FN | *S. aureus* |  |  | FN |  | FP | RLI | Missing |
| 123_1 | 123 | 1 | I | B | FN |  |  | Mixed skin | FN |  | FP | RLI | Missing |
| 123_2_a |  | 2a | I | B | FN |  |  | FN | FN |  | FN | RLI | Missing |
| 123_2_b |  | 2b | I | B | FN |  |  | Mixed skin | FN |  | FN | RLI | Missing |
| 123_3 |  | 3 | I | B | FN |  |  | Mixed skin | FN |  | FN | RLI | Missing |
| 123_4 |  | 4 | III | D | *Candida sp* |  | *Enterics* |  | FP | *C. parapsilosis*^†^ | FP | RLI | Missing |
| 124_1 | 124 | 1 | I | B | FN |  |  | Mixed skin | FN |  | FN | RLI | Healed |
| 125_1 | 125 | 1 | III | B | FN |  |  | Mixed skin | FN |  | FN | RLI | Still present but improved |
| 125_2 |  | 2 | III | D | FN |  |  | Mixed skin | FP | *C. parapsilosis^#^* | FN | RLI | Still present but improved |
| 125_3 |  | 3 | I | A | FN | *S. aureus, Enterococcus species* |  |  | FN |  | FN | RLI | Missing |
| 126_1 | 126 | 1 | II | A | FN |  |  | Mixed skin | FN |  | FP | RLI | Still present but improved |
| 127_1 | 127 | 1 | II | B | FN |  |  | FN | FN |  | FN | RLI | Still present but improved |
| 127_2 |  | 2 | II | B | FN |  |  | Mixed skin, Anaerobes | FN |  | FN | RLI | Missing |
| 127_3 |  | 3 | II | A | FN |  |  | Anaerobes, mixed skin | FN |  | FN | RLI | Missing |
| 128_1 | 128 | 1 | II | A | *C. albicans* |  | *Enterics* | Mixed skin | FN |  | FN | RLI | Healed |

**Table S2.** **Planktonic MIC (sMIC) and sessile MIC (sMIC) of fungal species isolated from wound swabs, against three conventional antifungals**: Fluconazole (FZ), Caspofungin (CAS) and Amphotericin B (AMB). Values represent median from three replicates. Growth inhibition was assessed visually, except for Fluconazole which were read by a spectrophotometer at 530 nm for 50 % inhibition due to the trailing effect. Symbols ^#^ and ^†^ indicate smooth and wrinkled phenotype of *C. parapsilosis* complex isolates respectively.

| **Patient** | **Fungi** | **PMIC (μg/mL)** | | |  | **SMIC at 90% (μg/mL)** | | |
| --- | --- | --- | --- | --- | --- | --- | --- | --- |
|  |  | **FZ** | **CAS** | **AMB** |  | **FZ** | **CAS** | **AMB** |
| 2_2 | *C. albicans* | 0.5 | 2 | 1 |  | >128 | 64 | 2 |
| 2_3 | *C. albicans* | 0.5 | 2 | 1 |  | >128 | 64 | 1 |
| 2_4 | *C. albicans* | ≤ 0.125 | 0.5 | ≤ 0.125 |  | >128 | 64 | 1 |
| 2_5 | *C. albicans* | ≤ 0.125 | 0.25 | ≤ 0.125 |  | >128 | 64 | 1 |
| 2_7 | *C. albicans* | 0.25 | 0.5 | ≤ 0.125 |  | >128 | 1 | 2 |
| 10_1 | *C. parapsilosis^#^* | 1 | 4 | 1 |  | >128 | >128 | 4 |
| 10_2 | *C. parapsilosis^#^* | 0.5 | 4 | 1 |  | 8 | >128 | 1 |
| 10_11_a | *C. parapsilosis^#^* | 2 | 1 | ≤ 0.125 |  | >128 | >128 | >128 |
| 17_2 | *C. glabrata* | 8 | 0.5 | 0.25 |  | >128 | >128 | >128 |
| 18_2 | *C. glabrata* | 4 | 0.5 | ≤ 0.125 |  | >128 | 2 | 8 |
| 18_4_b | *C. glabrata* | 4 | 0.5 | ≤ 0.125 |  | >128 | >128 | >128 |
| 22_3 | *R. mucilaginosa* | >64 | 16 | 0.5 |  | >128 | >128 | >128 |
| 24_2 | *C. parapsilosis*^†^ | 1 | 1 | ≤ 0.125 |  | >128 | >128 | 0.5 |
| 24_4 | *C. parapsilosis*^†^ | 2 | 4 | 1 |  | >128 | >128 | 64 |
| 28_4 | *C. parapsilosis*^†^ | 2 | 4 | 1 |  | >128 | >128 | 4 |
| 57_7_b | *C. albicans* | 0.25 | 0.25 | ≤ 0.125 |  | >128 | 64 | 2 |
| 71_1 | *C. parapsilosis ^#,^* ^†^ | 1^#^,2^†^ | 1 ^#, †^ | ≤ 0.125 ^#, †^ |  | >128 ^#, †^ | >128 ^#, †^ | 4^#^, 0.5^†^ |
| 85_3 | *C. parapsilosis^#^* | 1 | 4 | 1 |  | >128 | >128 | 16 |
| 101_1 | *C. parapsilosis^#^* | 1 | 1 | ≤ 0.125 |  | >128 | >128 | 32 |
| 102_1 | *C. parapsilosis^#^* | 1 | 1 | ≤ 0.125 |  | >128 | >128 | 64 |
| 102_3 | *C. parapsilosis*^†^ | 1 | 4 | 1 |  | >128 | >128 | >128 |
| 102_4 | *C. parapsilosis^#^* | 1 | 1 | ≤ 0.125 |  | >128 | >128 | 4 |
| 103_1 | *C. metapsilosis^#^* | 2 | 2 | 1 |  | >128 | >128 | 4 |
| 111_3 | *C. parapsilosis^#^* | 1 | 1 | ≤ 0.125 |  | >128 | >128 | 32 |
| 111_4 | *C. parapsilosis^#^* | 1 | 1 | ≤ 0.125 |  | >128 | >128 | >128 |
| 111_6 | *C. parapsilosis^#^* | 1 | 4 | 1 |  | >128 | >128 | >128 |
| 112_2_a | *C. parapsilosis^#^* | 1 | 1 | ≤ 0.125 |  | >128 | >128 | >128 |
| 112_3 | *C. parapsilosis^#^* | 1 | 1 | ≤ 0.125 |  | >128 | >128 | 2 |
| 120_1 | *C. albicans* | 1 | 2 | 1 |  | >128 | 1 | 4 |
| 123_4 | *C. parapsilosis*^†^ | 1 | 1 | ≤ 0.125 |  | >128 | >128 | 4 |
| 125_2 | *C. parapsilosis^#^* | 1 | 4 | 1 |  | >128 | >128 | 4 |
